# Late Testicular Yolk Sac Tumor With Cerebellar Metastasis and a Rapid Recurrence After a Gross Total Resection: A Case Report

**DOI:** 10.7759/cureus.17143

**Published:** 2021-08-13

**Authors:** Anna M Nia, Randall Z Allison, Megan Sweeney, Rudy P Briner

**Affiliations:** 1 Department of Neurosurgery, University of Texas Medical Branch, Galveston, USA

**Keywords:** late testicular yolk sac tumor relapse, non-seminoma germ cell tumor, conus medullaris, uncommon metastasis, suboccipital craniotomy, brain and lung metastasis

## Abstract

Yolk sac tumor (YST) is the most common prepubertal testicular tumor. It is considered a subtype of non-seminoma germ cell tumor (NSGCT) that is presumed to have an aggressive behavior with high malignant potential, thus requiring multimodality treatment with resection and chemotherapy. Treatment is curative for the majority of patients, even the ones with relapse after a few years. Here, we describe for the first time an atypical case of YST recurrence 17 years after primary treatment of YST. This is a case of YST in a 32-year-old man who presented with a large cerebellar mass consistent with YST recurrence after being in remission for 17 years. He underwent suboccipital craniotomy and complete excision of the tumor, as evident on postoperative MRI with a plan for stereotactic radiosurgery with dose and fractionation determined by MRI at four weeks postoperatively. However, the four-week MRI postoperatively revealed a large mass that was engulfing the prior resection cavity, indicative of unusual rapid tumor recurrence despite evidence of complete resection. The highly aggressive nature of this tumor should prompt clinicians to consider chemotherapy and radiation earlier than four weeks postoperatively.

## Introduction

Testicular germ cell tumors (GCTs) consist of seminoma and non-seminoma germ cell tumors (NSGCT). Endodermal sinus tumors or yolk sac tumors (YSTs), a type of NSGCT, are rare in adult patients, but they are the most common pediatric testicular tumors [1]. Microscopically, YSTs present with Schiller-Duval bodies presented as a central blood vessel surrounded by germ cells resulting in a glomerulus-shaped structure. Prognosis depends on the extent of the tumor and histology on presentation [2]. Overall, brain metastases in men with disseminated GCT are very rare [3]. In particular, only 1.3% of men with disseminated NSGCT had brain metastasis before they started chemotherapy [4]. A pooled analysis of 523 men with brain metastasis from GCT either at initial diagnosis or at relapse showed that high dose chemotherapy and multimodality treatment were associated with higher survival probabilities in men who had brain metastasis at relapse [5]. In general, the current management for NSGCT with brain metastasis is not optimized due to the rarity of afflicted patients thus far. In this report, we describe an unusual case of a 32-year-old male with a cerebellar metastatic yolk cell tumor undergoing gross total resection followed by recurrence and doubling in size of the tumor in just three weeks postoperatively.

## Case presentation

**Figure 1:**
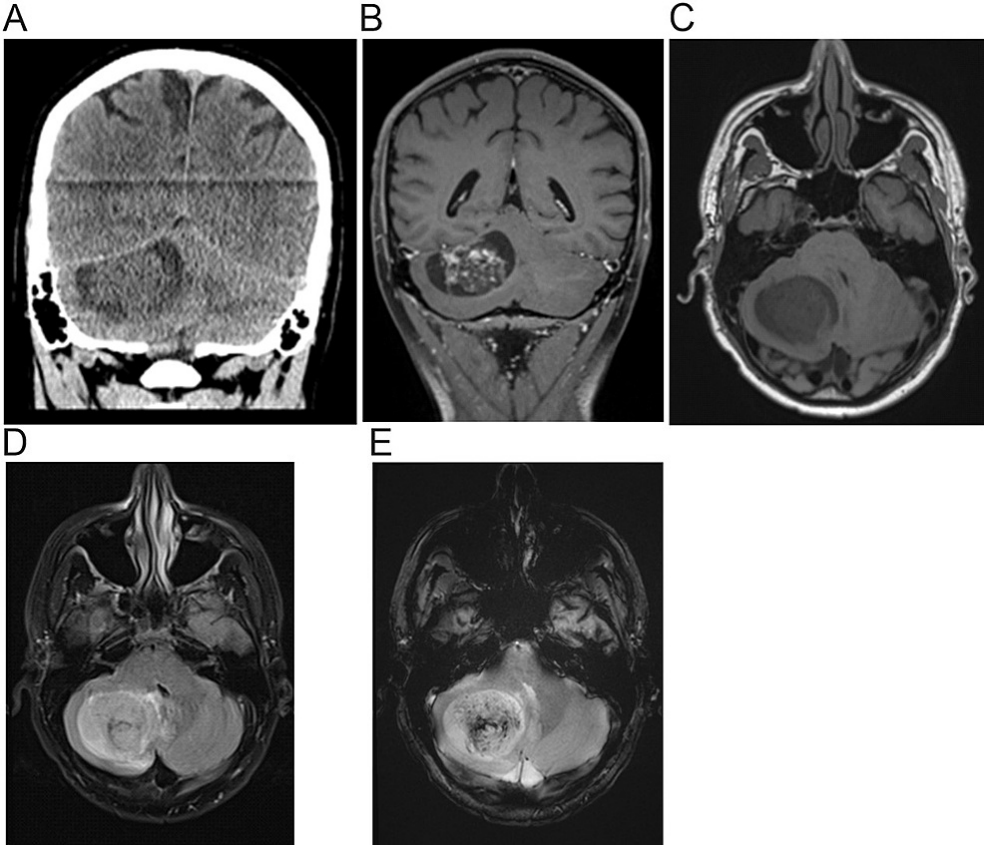
CT scan and MRI of the brain prior to craniotomy (A) CT scan of the head without contrast showing a 42 x 48 x 32 mm round right cerebellar mass with regional mass effect and effacement of the right paramedian cistern. A hemorrhage within the center of the mass is noted. Partial obliteration of the fourth ventricle without appreciable dilatation of the third and lateral ventricles as well as mild descent of the right cerebellar tonsil by about 3.5 mm. (B-E) MRI [(B): T1 (longitudinal relaxation time) with contrast, (C): T1 without contrast, (D): Fluid-attenuated inversion recovery (FLAIR), and (E): Gradient echo sequences (GRE)] of the brain showing a 49 x 4 x 37 mm cystic, solid enhancing hemorrhagic right cerebellar mass with local mass effect with partial effacement of the fourth ventricle, mass effect on the right posterior brainstem, and mild inferior protrusion of right cerebellar tonsil below the foramen magnum. No hydrocephalus.

A 32-year-old man presented to the emergency department with a two-day history of worsening headaches, dizziness, some vision changes, dysarthria, and memory disturbances. On admission, motor examination revealed right upper extremity dysmetria with no weakness or numbness and no cranial nerve palsies. He had a past medical history of YST of the right testes diagnosed at the age of 15. He was treated with right orchiectomy and retroperitoneal lymph node dissection (RPLND) as well as four cycles of BEP [bleomycin, etoposide, and Platinol (cisplatin)] and was in remission for 17 years. He presented to the emergency department five months prior to this admission with acute hypoxic respiratory distress and was then diagnosed with YST metastasis to the left hilar/posterior mediastinal and spleen. Subsequently, he was treated with four cycles of VIP [Vepesid (etoposide), ifosfamide, and Platinol (cisplatin)] with ~30% treatment response consistent with RECIST (Response Evaluation Criteria in Solid Tumors), partial response and normalized alpha-fetoprotein (AFP) and beta-human chorionic gonadotropin (ß-hCG). On this admission, a computed tomography (CT) scan of the head without contrast revealed a large right cerebellar mass (Figure 1A). Magnetic resonance imaging (MRI) with contrast studies confirmed a hemorrhagic cystic, solid enhancing right cerebellar mass (Figure 1B-1E).

**Figure 2:**
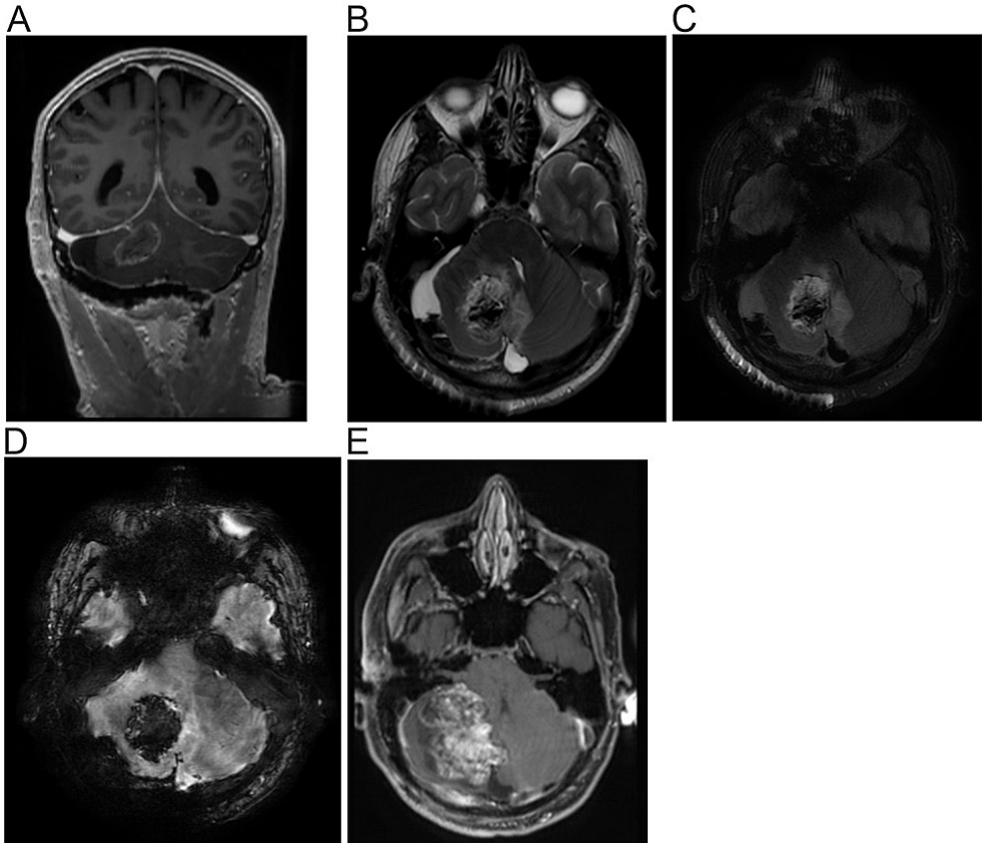
MRI of the brain one day and four weeks post craniotomy (A-D) MRI [(A): T1 (longitudinal relaxation time) without contrast, (B): T2 (transverse relaxation time), C: Fluid-attenuated inversion recovery (FLAIR), and D: Susceptibility-weighted angiography (SWAN)] of the brain post craniotomy with EVD. Postsurgical changes of right suboccipital craniotomy and resection of the right cerebellar tumor. Blood present within the resection cavity and at the periphery limited evaluation for enhancement. However, there was no evidence of intra- or perilesional enhancement to suggest residual disease. (E) MRI of the brain with contrast four weeks post craniotomy. A mixed solid and cystic mass in the right cerebellar hemisphere with a heterogeneous enhancement of the solid component that measures approximately 63 x 52 x 34 mm engulfing the prior resection cavity. The lesion extends to the left cerebellar hemisphere and causes a mass effect on the right side of the brainstem, and results in effacement of the fourth ventricle. The mild dilatation of the lateral and third ventricles is suggestive of developing hydrocephalus.

The patient underwent right frontal external ventricular drain (EVD) placement and a suboccipital craniotomy for transcerebellar tumor resection. The tumor was noted as very friable and was easily suctioned from the surrounding parenchyma. The friable tumor was completely removed with no intraoperative nor postoperative complications. Postoperative MRI with and without gadolinium of the brain revealed no evidence of enhancement to suggest residual disease (Figure 2A-2D). 

Pathological examination of the tumor specimen confirmed a metastatic malignant germ cell tumor consistent with his known prior primary diagnosis of YST. Postoperatively, AFP was elevated at 68.3 ng/mL, and ß-hCG was within the normal range, findings consistent with a YST. The patient was evaluated for the metastatic source of the brain lesion with full-body CT, which revealed a splenic hypodense lesion, measuring 1.8 cm previously noted to be 3.2 cm three months prior to the operation. The previously found left hilar mass noted five months prior was unchanged in size. There were no new intra-abdominal/pelvic metastases nor lymphadenopathy. The EVD was removed postoperatively, and he was subsequently discharged home after an uneventful postoperative course.

He was evaluated three weeks later as an outpatient for consideration of radiation therapy. He stated he was recovering well and denied vision changes, focal weakness, focal sensory loss, pain, and bowel/bladder incontinence. Postoperative stereotactic radiosurgery was recommended with dose and fractionation determined after MRI, but MRI at four weeks postoperatively showed a large heterogeneously enhancing solid and cystic mass in the right cerebellar hemisphere engulfing the prior resection cavity. (Figure 2E) A repeat serum AFP was 485 ng/mL with normal serum ß-hCG. Due to concern for a rapidly developing hydrocephalus, the patient was subsequently admitted for a redo suboccipital craniotomy and tumor resection. The tumor was again noted to be very friable and was completely removed with no complications. Surgical pathology showed morphologically identical tumor specimen consistent with the patient’s known history of YST. Immunohistochemistry of the tumor specimen was AFP and Glypican3-positive. No other germ cell tumor components were identified in the resected tissue.

Postoperative MRI of the neuroaxis revealed new metastasis in the cervical spine and an enhancing lesion within the conus medullaris, findings reflective of drop metastasis. Due to the rapid development of his tumor, he began radiation therapy one week postoperatively, a total dose of 40 Gy of focal external beam radiation to the cerebellum and spinal lesions in 10 fractions (400 cGy/fraction), over 14 days using 6 MV photons via intensity-modulated radiation therapy. He tolerated the radiation well with no side effects but was subsequently admitted six weeks postoperatively for back pain and a urinary tract infection. An updated MRI of the cervical and lumbar spine revealed significant progression of the disease with a large conglomerate of metastasis at T12-L1 vertebra. On admission, his serum AFP was 421 ng/mL. The patient’s Karnofsky performance score continued to decline due to progressive bilateral lower extremities and left upper extremity weakness. He was discharged home with plans for hospice care and palliative spine radiation therapy as an outpatient and passed away two months later.

## Discussion

The rate of NSGCT late relapse (> two years) after successful primary treatment is estimated to be 1-3% [6]. However, most studies for late relapses are limited by lack of long-term follow-up (>two years), lack of subsequent clinical outcomes for patients undergoing surveillance, or losing patients to follow-up after five years. The aforementioned patient presented with a late relapse to lung and spleen, followed by brain and spine 17 years after primary YST treatment with orchiectomy with RPLND and BEP chemotherapy. This resulted in a fatal case 17 years later, which is rare in treated primary YST cases.

The optimal management of patients, especially in the pediatric population with YST of the testis, remains controversial. The major controversies are the use of chemotherapy [BEP/etoposide and cisplatin (EP)] with or without RPLND as well as radiation therapy. The present case highlights the importance of long, even lifelong follow-up, although RPLND has been shown to be effective in preventing metastasis of YST in some cases [7-8]. This patient did not have an annual surveillance CT. The presented case suggests that patients with metastasis of YST to the brain at presentation are likely to have a poor prognosis even with gross total resection. Urgent radiotherapy of the resection cavity sooner than our standard four-week timeframe may have provided some benefit by halting tumor progression. Consequently, it is of utmost importance to provide information about annual and life-long surveillance in patients with a history of YST. Annual scans are cost prohibited and are seen as a burden to the patients and the hospital systems, but the risk of cancer relapse with metastasis may outweigh the perceived burden. Several studies support lifelong follow-up and surveillance in patients with testicular cancer [9-10].

## Conclusions

Though rare, intracranial YST metastasis has an abysmal prognosis. Therefore, a combination of surgical resection, chemotherapy, and radiotherapy is often recommended for the management. The presented case showed that YST brain metastasis could have an unusually rapid recurrence despite a gross total resection. Due to its high propensity to recur and metastasize to the spine, if feasible, the craniotomy for complete resection of the tumor should be followed urgently with radiation therapy and chemotherapy before four weeks postoperatively. This approach in special cases with high metastatic potential may be more effective at preventing cerebrospinal fluid (CSF) dissemination, further metastasis, and multifocal disease. Nonetheless, given the highly aggressive potential of YST with central nervous system (CNS) presentation, the most effective approach is long-term and regular surveillance to diagnose possible YST recurrence as early as possible before evidence of CNS metastasis.
